# Six-year changes in refraction and related ocular biometric factors in an adult Chinese population

**DOI:** 10.1371/journal.pone.0183364

**Published:** 2017-08-30

**Authors:** Xiaotong Han, Xinxing Guo, Pei Ying Lee, Ian G. Morgan, Mingguang He

**Affiliations:** 1 State Key Laboratory of Ophthalmology, Zhongshan Ophthalmic Center, Sun Yat-sen University, Guangzhou, China; 2 Centre for Eye Research Australia; Ophthalmology, Department of Surgery, University of Melbourne, Melbourne, Australia; 3 ARC Centre of Excellence in Vision Science and Visual Sciences Group, Research School of Biology, College of Medicine, Biology and Environment, Australian National University, Canberra, Australia; Soochow University Medical College, CHINA

## Abstract

**Purpose:**

To investigate longitudinal changes in refraction and biometry in Chinese adults.

**Design:**

Population-based prospective cohort study.

**Methods:**

1817 subjects aged ≥ 35 years were randomly recruited from Yuexiu district, Guangzhou, China in 2008. Of which 1595 (87.8%) were reexamined in 2010 and 1427 (78.5%) were reexamined in 2014. Non-cycloplegic automated refraction and visual acuity test were performed at baseline and the 6-year follow-up examination for all participants. In addition, 50% of the participants were randomly selected for axial length (AL), anterior chamber depth (ACD) and lens thickness (LT) measurements using non-contact partial coherence laser interferometry. Lens power (LP) was calculated with the Bennett’s equation.

**Results:**

A total of 1300 participants were included in current analysis (2008 mean [SD] age, 51.4 [10.6] years; 54.5% women). Mean change in spherical equivalence (SE) was +0.24 (95% confidence interval [CI], +0.19 to +0.30), +0.51 (95% CI, +0.46 to +0.57), +0.26 (95% CI, +0.15 to +0.38) and -0.05 (95% CI, -0.21 to +0.10) diopters (D) for individuals in the age groups of 35 to 44, 45 to 54, 55 to 64 and 65+ years at baseline, respectively. Corneal power, AL and LT increased while ACD and LP decreased during the follow-up. Baseline SE and changes in biometric factors could explain 97.2% of the variance in longitudinal SE change while LP solely could explain 65.2%. Six-year mean change in cylinder power was -0.16 (95% CI, -0.19 to -0.13) D, the axis of astigmatism changed from “with-the-rule” to “against-the-rule” in 16.4% of the participants and to “oblique” in 0.9%.

**Conclusions:**

This study confirms a hyperopic shift in the elderly before 65 years old and a myopic shift thereafter. Longitudinal refraction change could be well explained by corresponding biometry changes, especially LP. There is also a shift to “against-the-rule” astigmatism for the adult population.

## Introduction

Uncorrected refractive error is the most common cause of moderate or severe vision impairment and the second leading cause of blindness globally.[1] The prevalence of refractive error, especially myopia, is high and will continue to increase in the following decades in East Asia population.[2–4] Previous studies have illustrated that refraction changes continued into older adulthood.[5, 6] Thus a better understanding of refractive changes in adults and its associated factors could help to better estimate the eye care need in the aging society. In addition, the ever-growing popularity of refractive surgery also calls for a better understanding of refraction and biometric change in adulthood to predict and monitor long-term surgical outcome.

Cross-sectional studies on the prevalence of refractive error in adulthood reported an overall decreasing trend for myopia and an increasing trend for hyperopia with age.[7–10] However, it is unclear whether this apparent reducing pattern on myopia was due to more myopic people in the younger age group (cohort effect) or an authentic hyperopic shift with aging.

Longitudinal studies on refractive change were mostly focused on children as they had the most rapid change. The natural history of refractive errors among older adults was not well understood until recently. The Beaver Dam Eye Study (BDES), Blue Mountain Eye Study (BMES), Barbados Eye Study (BES) and Reykjavik Eye Study all reported significant longitudinal changes in refraction in adults with younger people becoming more hyperopic and older people more myopic, although the specific ages for the beginning and end of hyperopic shift differed across studies.[11–14] However these studies did not collect prospective ocular biometric data and were not able to assess the accompanying longitudinal biometric changes and its association with refraction changes.

The Shahroud Eye Cohort Study is the only study, reporting a 5-year longitudinal change in refraction and ocular components in an Iranian population, however the age range was limited to 40 to 64 years old and the refraction data measurement were done differently at baseline (with cycloplegia) and follow-ups (without cycloplegia).[15] We previously identified a 2-year hyperopic shift with aging in an adult Chinese population with some evidence suggesting the existence of cohort effects, but the 2-year follow-up period is too short to estimate the longitudinal refraction or biometric change.[16] Thus the purpose of this study was to investigate the 6-year change in refractive error and related biometric factors in the same population to better address this issue.

## Materials and methods

### Study population

Random cluster sampling was used to identify participants aged 35 years or older from Yuexiu District, Guangzhou, China, in December 2008. Details of the recruitment and study methodology have been reported elsewhere.[17] Briefly, 1817 of 2284 (79.6%) eligible subjects were enrolled at baseline and personal information such as name, age and gender, were collected. All participants were invited for the 2-year and 6-year follow-up examinations in 2010 and 2014, respectively. Of the 1817 participants examined at baseline, 1427 (78.5%) were reexamined in 2014. Follow-up examinations took place at Zhongshan Ophthalmic Center (ZOC) in Guangzhou or in local community facilities or homes for individuals with mobility restrictions or limited free time. Same procedures and protocols were applied throughout the study. The main protocol was approved by the World Health Organization Secretariat Committee on Research Involving Human Subjects and by the institutional review board at ZOC in Guangzhou, China. Approval for the follow-up survey was also obtained from the responsible institutional review ethics committee. Written informed consent was obtained from all participants and the study was conducted in accordance with the tenets of the Declaration of Helsinki. The participants did not receive any financial compensation.

### Procedures

Questionnaires including information on education background and detailed ophthalmic surgical history were administered by trained nurses at baseline and both follow-ups (S1 File). Distance visual acuity was measured indoor under ambient lighting with a LogMAR ETDRS tumbling E chart (Precision Vision, La Salle, Illinois, USA) per standardized protocol. Participants with uncorrected binocular visual acuity of ≤ 20/40 were further tested with subjective refraction to obtain their best-corrected visual acuity (BCVA) at the 2014 follow-up. Non-cycloplegic automated refraction was carried out for all participants at baseline and at each follow-up using the same device after proper calibration (KR-8800; Topcon Corp). Five consecutive measurements were performed for each eye with the mean recorded as the final value. Corneal refractive power was measured using the same autorefractor and the mean of five readings was recorded for each eye. Slit-lamp examination was performed by an ophthalmologist to assess the anterior segment. The presence or absence of cataract was also determined during slit-lamp examination, based on the assessment of light red reflex on the lens.

Half of the participants who were examined during baseline visit were subsequently selected, via systematic random sampling, to undergo detailed biometric examination on the same visit. Follow-up biometric examinations were only carried out in the same group of participants using identical equipment and protocol.

Axial length (AL) and anterior chamber depth (ACD) were measured separately in each eye using non-contact partial coherence laser interferometry (IOLMaster, version 3.0, Carl Zeiss Meditec at baseline; Lenstar LS900, Haag-Streit AG, Switzerland at the 2014 follow-up; and both at the 2010 follow-up) in a dark room (illumination < 5 lux). Measurements with a ratio of signal to noise less than 2.0 or those that differed from other values by more than 0.1 mm were re-measured. The mean of 5 measurements was recorded as the final result.

Anterior segment optical coherence tomography (AS-OCT) imaging (Visante; Carl Zeiss Meditec) was performed with one scan centered over the pupil taken on the horizontal meridian (0°-180°) in a dark room at baseline and the 2010 follow-up. The image of each eye with the best quality (based on visibility of the scleral spur and the maximum interference flare) was selected. Custom software (ZAAP; Zhongshan Angle Assessment Program) was used to perform noise and contrast conditioning on all images. The borders of the corneal epithelium and endothelium, the anterior and the posterior surface of the iris and the anterior surface of the lens were automatically defined. An observer manually define the nasal and temporal scleral spur on each scan and the software can automatically calculate the lens thickness (LT), defined as the shortest distance between the anterior and posterior poles of the lens. LT was measured by AS-OCT at baseline, by noncontact partial coherence laser interferometry (Lenstar LS900, Haag-Streit AG, Switzerland) in 2014, and by both in 2010.

### Data management and analyses

Spherical equivalent (SE), calculated as spherical power plus half of cylindrical power, was used to represent refraction. Change in refraction was defined as the SE at baseline subtracted from the SE at the 2014 follow-up. Similarly, changes in biometric factors were defined as the corresponding values at baseline subtracted from the values at the 2014 follow-up. Hyperopia was defined as SE >+0.5 diopters (D), mild myopia was defined as -3D ≤ SE <-0.5 D and moderate to high myopia was defined as SE <-3D. Baseline refractive state was categorized into moderate to high myopia, mild myopia, emmetropia and hyperopia according to the above definitions, with a corresponding sample size of 131, 256, 550 and 363, respectively. A myopic shift was defined as a negative longitudinal change in SE whereas a hyperopic shift was defined as a positive change. Age was categorized into four age groups: 35–44, 45–54, 55–64 and ≥65, based on the age obtained at baseline, and the corresponding sample size was 368, 498, 273 and 161, respectively. Education level was dichotomized as less than high school and high school or above, which include 414 and 886 subjects, respectively. Participants who had undergone refractive surgeries or were aphakic or pseudophakic were exclude from the analysis. Eyes with BCVA ≤20/200 were also excluded because of increased variation in refraction measurements.

There was a strong correlation between SE of the left and right eyes, thus only data from right eyes were presented. The AL data measured by IOLMaster correlated well with the measurement obtained using Lenstar in 2010 (linear regression β = 0.996), therefore an equation is generated to relate these values. This allowed us to convert the AL data measured by Lenstar in 2014 into equivalent IOLMaster value for comparison with the baseline AL data measured by IOLMaster. A same approach was performed for the comparison of ACD measurements (linear regression β = 0.923). Similarly, as the LT data measured by AS-OCT and Lenstar in 2010 also correlated well (linear regression β = 1.286), the LT data measured by Lenstar in 2014 was converted into equivalent AS-OCT values based on the equation generated from the LT data in 2010 for comparison of LT data collected in 2014 with the baseline LT data. Lens power was calculated according to the Bennett’s equation based on refractive error and biometric parameters.[18]

All statistical analyses were performed using STATA Statistical Software: Release 12.0 (StataCorp LP, Colleage Station, TX). Independent two-sample t-test was used to assess the difference between participants who were included and excluded from the current analysis. T-test and trend-analysis were used to assess the difference in the 6-year changes of refractive and biometric factors between different age groups, gender, education background and baseline SE status. Univariate and global search regression (GSREG) were used to model the effects of various variables including longitudinal changes of biometric factors on the 6-year refraction change. GSREG is a new automatic model selection technique for multiple regression, and all the variables in univariate analysis were included in our GSTEG analysis.[19] P-values of <0.05 were considered statistically significant.

## Results

Of the 1427 participants at the 2014 follow-up, we further excluded 44 (3.1%) participants whose refraction data were unavailable at baseline or the 2014 follow-up, 82 (5.7%) who had undergone corneal or lens surgeries and 1 (0.7%) whose BCVA was worse than 20/200 at 2014 follow-up. The remaining 1300 (91.1%) participants were included in the current analysis with a mean age of 51.4 ± 10.6 years and 54.5% were female. Those included in the analysis were significantly younger (P<0.001), with a higher education level (P<0.001) and smaller AL (P = 0.03), as well as less cataract (P<0.001) (S1 Table).

Table 1 presents the 6-year changes in SE and related ocular biometric factors. An overall hyperopia shift of +0.31 D (95% confidence interval [CI]: 0.27 to 0.36) was identified for the population under study. For participants aged 35 to 44, 45 to 54, 55 to 64 and 65+ years at baseline, the corresponding SE change was +0.24 D (95% CI: 0.19 to 0.30), +0.51 D (95% CI: 0.46 to 0.57), +0.26 D (95% CI: 0.15 to 0.38) and -0.05 D (95% CI: -0.21 to 0.10), respectively. Participants with higher educational level had larger hyperopic shift (P<0.05) and myopic participants had smaller hyperopic shift (P<0.001). Corneal power increased during the follow-up with a mean change of +0.28 D (95% CI: 0.26 to 0.29), and was more significant in younger participants (P<0.001). The change in corneal power did not differ by gender, education or baseline refractive state. AL increased by 0.07mm (95%CI: 0.05 to 0.10) and was more significant in older subjects (P<0.001) and myopes (P<0.05). Changes in ACD and LT were -0.06mm (95%CI: -0.07 to -0.05) and 0.15mm (95%CI: 0.13 to 0.16), respectively. Both younger and more myopic participants had greater decrease in ACD and increase in LT (P<0.001). Subgroup analysis showed that the change in ACD is roughly half of the change in LT. The mean change in LP was -1.62 D (95%CI: -1.72 to -1.52) and was more significant in younger (P<0.001) and more highly educated participants (P<0.05). A significant birth cohort effect on SE change was also identified that younger cohorts, among those aged 35–40 years, were more myopic compared to older cohorts when they were at the same age, especially in the youngest age group (S1 Fig).

**Table 1:** Six-year changes of spherical equivalence and related biometric factors in the right eye.

Characteristic	SE (mean, 95% CI),D	Corneal power (mean, 95% CI),D	AL (mean, 95% CI),mm	ACD (mean, 95% CI),mm	LT (mean, 95% CI),mm	LP (mean, 95% CI),D
Total No.	1300	1273	598	596	566	554
Difference	0.31(0.27 to 0.36)	0.28(0.26 to 0.29)	0.07(0.05 to 0.10)	-0.06(-0.07 to -0.05)	0.15(0.13 to 0.16)	-1.62 (-1.72 to -1.52)
**Age group**						
35–44	0.24(0.19 to 0.30)	0.30(0.27 to 0.34)	0.06(0.01 to 0.11)	-0.09(-0.11 to -0.08)	0.22(0.19 to 0.24)	-1.78(-1.94 to -1.62)
45–54	0.51(0.46 to 0.57)	0.29(0.26 to 0.31)	0.06(0.04 to 0.09)	-0.05(-0.07 to -0.03)	0.14(0.12 to 0.17)	-1.93 (-2.02 to -1.84)
55–64	0.26(0.15 to 0.38)	0.24(0.20 to 0.27)	0.08(0.06 to 0.11)	-0.07(-0.09 to -0.05)	0.11(0.09 to 0.13)	-1.24 (-1.51 to -0.98)
≥65	-0.05(-0.21 to 0.10)	0.26(0.20 to 0.32)^a^	0.12(-0.00 to 0.24)^b^	-0.00(-0.04 to 0.03)^b^	0.08(0.01 to 0.16)^b^	-0.89 (-1.41 to -0.36)^b^
**Sex**						
Male	0.34(0.28 to 0.41)	0.28(0.25 to 0.30)	0.08(0.04 to 0.11)	-0.06(-0.08 to -0.05)	0.16(0.14 to 0.17)	-1.69 (-1.84 to -1.53)
Female	0.29(0.23 to 0.34)	0.28(0.25 to 0.30)	0.07(0.05 to 0.10)	-0.06(-0.08 to -0.05)	0.14(0.12 to 0.17)	-1.57 (-1.70 to -1.43)
**Education**						
Less than high school	0.25(0.17 to 0.32)	0.28(0.25 to 0.31)	0.10(0.05 to 0.15)	-0.05(-0.07 to -0.03)	0.10(0.07 to 0.14)	-1.41 (-1.63 to -1.19)
High school or above	0.35(0.30 to 0.40)^a^	0.28(0.25 to 0.30)	0.06(0.04 to 0.08)	-0.07(-0.08 to -0.06)	0.17(0.15 to 0.18)^b^	-1.72 (-1.83 to -1.61)^a^
**Baseline refractive state**						
Moderate to high myopia	0.01(-0.19 to 0.22)	0.23(0.12 to 0.35)	0.27(0.07 to 0.46)	-0.08(-0.12 to -0.04)	0.16(0.09 to 0.24)	-1.77 (-2.38 to -1.16)
Mild myopia	0.18(0.06 to 0.29)	0.30(0.27 to 0.33)	0.09(0.04 to 0.14)	-0.07(-0.11 to -0.03)	0.17(0.14 to 0.20)	-1.35 (-1.65 to -1.05)
Emmetropia	0.41(0.36 to 0.45)	0.28(0.26 to 0.31)	0.04(0.03 to 0.05)	-0.07(-0.08 to -0.06)	0.16(0.13 to 0.18)	-1.76 (-1.85 to -1.67)
Hyperopia	0.38(0.31 to 0.45)^b^	0.27(0.24 to 0.30)	0.04(0.02 to 0.06)^a^	-0.04(-0.06 to -0.02)^b^	0.12(0.10 to 0.14)^b^	-1.55 (-1.74 to -1.36)

SE: spherical equivalence; AL: axial length; ACD: anterior chamber depth; LT: lens thickness; LP: lens power; D: diopter; CI: confidence interval.

^a^ P < 0.05;

^b^ P < 0.001;

P values were results of P-trend analysis.

Table 2 shows the 6-year changes of SE and related biometric factors for participants without cataract at both baseline and follow-ups. All subjects in this subgroup had hyperopic shift during the follow-up which was more significant in older subjects and hyperopes (P<0.001). The overall increase in LT as well as decrease in ACD and LP were larger than those observed in the total population.

**Table 2:** Six-year changes of spherical equivalence and related biometric factors in the right eye of participants without cataract at both baseline and follow-ups.

Characteristic	SE (mean, 95% CI),D	Corneal power (mean, 95% CI),D	AL (mean, 95% CI),mm	ACD (mean, 95% CI),mm	LT (mean, 95% CI),mm	LP (mean, 95% CI),D
Total No.	822	814	386	386	366	359
Difference	0.42(0.38 to 0.46)	0.30(0.28 to 0.32)	0.05(0.03 to 0.07)	-0.07(-0.08 to -0.06)	0.17(0.16 to 0.19)	-1.86(-1.94 to -1.78)
**Age group**						
35–44	0.25(0.20 to 0.31)	0.32(0.28 to 0.35)	0.06(0.01 to 0.11)	-0.09(-0.11 to -0.08)	0.22(0.19 to 0.24)	-1.79(-1.95 to -1.63)
45–54	0.55(0.49 to 0.60)	0.29(0.26 to 0.32)	0.04(0.03 to 0.06)	-0.05(-0.07 to -0.03)	0.16(0.14 to 0.17)	-1.95(-2.04 to -1.86)
55–64	0.53(0.44 to 0.61)	0.28(0.23 to 0.33)	0.04(0.01 to 0.07)	-0.06(-0.10 to -0.02)	0.12(0.09 to 0.15)	-1.69 (-1.93 to -1.45)
≥65	0.79(0.61 to 0.97)^b^	0.17(0.19 to 0.53)	0.05^a^	-0.15^a^	0.20(-0.17 to 0.57)^b^	-2.02
**Sex**						
Male	0.43(0.38 to 0.49)	0.29(0.26 to 0.32)	0.05(0.01 to 0.09)	-0.07(-0.08 to -0.05)	0.18(0.16 to 0.20)	-1.86(-1.99 to -1.73)
Female	0.41(0.36 to 0.46)	0.28(0.25 to 0.31)	0.05(0.02 to 0.08)	-0.07(-0.09 to -0.05)	0.18(0.16 to 0.20)	-1.87(-1.97 to -1.77)
**Education**						
Less than high school	0.47(0.40 to 0.54)	0.30(0.27 to 0.33)	0.05(0.03 to 0.07)	-0.07(-0.09 to -0.04)	0.16(0.14 to 0.18)	-1.85(-1.99 to -1.72)
High school or above	0.41 (0.36 to 0.45)	0.29(0.26 to 0.32)	0.05(0.02 to 0.08)	-0.07(-0.08 to -0.05)	0.18(0.16 to 0.20)	-1.87(-1.96 to -1.77)
**Baseline refractive state**						
Moderate to high myopia	0.16(-0.06 to 0.37)	0.25(0.11 to 0.39)	0.14(-0.03 to 0.30)	-0.11(-0.15 to -0.07)	0.23(0.20 to 0.26)	-1.93(-2.47 to -1.38)
Mild myopia	0.30(0.23 to 0.38)	0.31(0.27 to 0.34)	0.08(0.03 to 0.14)	-0.07(-0.12 to -0.03)	0.20(0.17 to 0.22)	-1.74(-1.90 to -1.57)
Emmetropia	0.46(0.42 to 0.49)	0.30(0.28 to 0.32)	0.03(0.02 to 0.05)	-0.07(-0.08 to -0.06)	0.17(0.15 to 0.19)	-1.83(-1.93 to -1.74)
Hyperopia	0.63(0.57 to 0.70)^b^	0.31(0.27 to 0.35)^a^	0.01(-0.00 to 0.03)	-0.03(-0.06 to -0.01)^b^	0.14(0.12 to 0.16)^b^	-2.06(-2.23 to -1.89)^b^

SE: spherical equivalence; AL: axial length; ACD: anterior chamber depth; LT: lens thickness; LP: lens power; D: diopter; CI: confidence interval.

^a^ P < 0.05;

^b^ P < 0.001

Table 3 shows the distribution of SE change by baseline age and gender. For participants with cataract, 13.9% had a myopic change of more than -0.5 D and 32.3% had a hyperopic change of more than 0.5 D. In contrast, for participants without cataract, the corresponding value was 4.8% and 38.6% respectively. With increasing baseline age, there is a myopic shift for both men and women.

**Table 3:** Distribution of mean change in spherical equivalence by baseline age and gender.

	With cataract (%)				Without cataract (%)			
**Women**	<-0.5D	-0.5D to 0.5D	>+0.5D	P	<-0.5D	-0.5D to +0.5D	>+0.5D	P
35–44	-	-	-	0.014	6.3	68.6	25.1	<0.001
45–54	0	62.1	37.9		1.0	49.5	49.5	
55–64	5.6	53.5	40.9		3.3	60.7	36.0	
≥65	21.4	50.0	28.6		28.6	57.1	14.3	
All ages	17.1	53.6	29.3		5.1	56.1	38.8	
**Men**								
35–44	-	-	-	0.019	7.3	67.3	25.4	<0.001
45–54	0	60.6	39.4		1.0	48.5	50.5	
55–64	6.7	54.7	38.6		3.2	61.3	35.5	
≥65	21.1	49.1	29.8		28.6	57.1	14.3	
All ages	10.3	53.9	35.8		4.5	57.3	38.2	
**Women and Men**								
35–44	-	100.0	-	<0.001	6.3	68.9	24.8	<0.001
45–54	1.5	53.0	45.5		1.6	48.2	50.2	
55–64	10.0	55.0	35.0		6.8	52.6	40.6	
≥65	23.9	52.2	23.9		30.4	43.5	26.1	
All ages	13.9	53.8	32.3		4.8	56.6	38.6	

D: diopter.

P values were results of P-trend analysis.

Table 4 shows the association between longitudinal changes in SE and related risk factors by linear regression model. More myopic baseline SE, decreasing corneal power, AL, LT and LP (P<0.001) were all significantly related to a hyperopic shift in SE. Baseline cataract (P<0.001) were associated with SE change in univariate analysis but not in multivariate analysis. In the multiple regression model, baseline SE and changes in biometric factors could explain 97.23% of variance in the longitudinal change of refraction. While changes in LP solely could explain 65.2% of the variation in univariate analyses.

**Table 4:** Linear regression models predicting 6-year changes in spherical equivalence.

Factors	Univariate regression			Multiple regression	
	Regression coefficient Mean(95% CI)	P Value	Adjusted R^2^	Regression coefficient Mean(95% CI)	P Value
Age, y	-0.009(-0.013 to -0.005)	<0.001	0.015	-	-
Sex, female	-0.06(-0.14 to 0.03)	0.18	0.001	-	-
Educational level, High school or above	0.10(0.01 to 0.19)	0.03	0.003	-	-
Baseline SE, D	0.06(0.04 to 0.07)	<0.001	0.026	-0.01(-0.02 to -0.01)	<0.001
ΔAL, mm	-0.25(-0.46 to -0.04)	0.02	0.007	-1.34(-1.38 to -1.29)	<0.001
ΔACD, mm	-0.12(-0.55 to 0.31)	0.59	0.001	-	-
ΔCorneal power, D	-0.13(-0.25 to -0.00)	0.05	0.002	-0.99(-1.03 to -0.95)	<0.001
ΔLT, mm	0.17 (-0.17 to 0.51)	0.34	0.001	-1.42(-1.48 to -1.36)	<0.001
ΔLP, D	-0.48(-0.50 to -0.45)	<0.001	0.652	-0.64(-0.65 to -0.63)	<0.001
Baseline cataract, yes	-0.23(-0.32 to -0.14)	<0.001	0.017	-	-

SE: spherical equivalence; AL: axial length; ACD: anterior chamber depth; LT: lens thickness; LP: lens power; D: diopter; CI: confidence interval.

The adjusted R2 values for the multiple regression model was 0.97.

**Fig 1:**
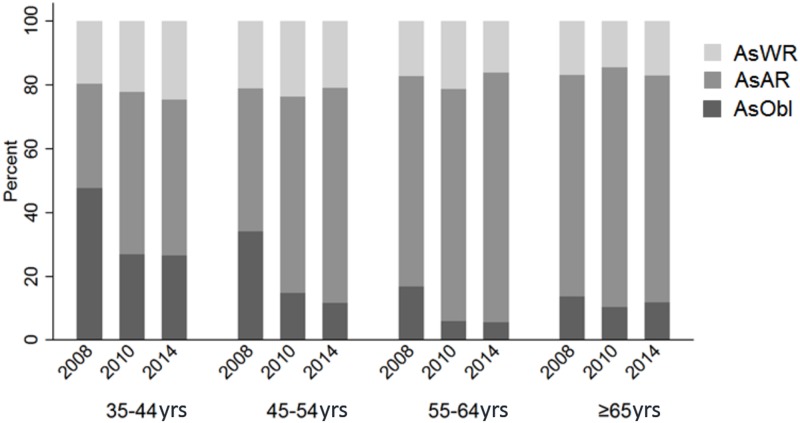
Distribution of astigmatism types at each examination in different age groups.

Table 5 shows the 6-year change of cylinder power by baseline age and gender. The overall change was -0.16 D (95%CI: -0.19 to -0.13) with older participants had a larger decrease (P<0.001) and no significant gender difference. Cylinder power decreased by more than -0.5 D in 11.01% of the participants and increased by more than 0.5 D in 2.31%. In terms of the axis of astigmatism, as shown in Fig 1 that the proportions of participants with “with-the-rule”, “against-the-rule” and “oblique” astigmatism were 31.54%, 49.08% and 19.38% respectively at baseline. During the 6-year follow-up, 16.4% of these cases changed to “against-the-rule” and 0.9% to “oblique” from “with-the-rule” astigmatism.

**Table 5:** Six-year changes of cylinder power.

	Change in Cylinder Power (D)		Change in Diopters (%)		
	N	Mean (95% CI)	<-0.5	-0.5 to +0.5	>0.5
**Age group**					
35–44	368	-0.10(-0.14 to -0.06)	3.42	95.44	1.14
45–54	498	-0.12(-0.19 to -0.06)	7.71	91.04	1.25
55–64	273	-0.26(-0.32 to -0.21)	15.47	83.02	1.51
≥65	161	-0.24(-0.37 to -0.12)*	30.57	59.88	9.55*
**Sex**					
Male	591	-0.17(-0.21 to -0.13)	10.09	87.61	2.30
Female	709	-0.15(-0.20 to -0.10)	11.77	85.90	2.33
**Total**	1300	-0.16(-0.19 to -0.13)	11.01	86.68	2.31

CI: confidence interval; D: diopter.

* P<0.001

## Discussion

The present study identified an overall +0.31D hyperopic shift over 6-years in this adult Chinese population with a significant hyperopic shift in participants under 65 years old and a myopic shift thereafter, as well as a notable longitudinal change in the degree and axis of astigmatism. We also illustrated that longitudinal refraction change could be largely explained by changes in biometric factors, especially LP. Strengths of the current study include a population-based design and high follow-up rate.

Population-based cross-sectional studies have reported hyperopic SE shift starting at around 40 years old, followed by myopic shift at around 65–75 years old.[9, 20] This trend was confirmed by several longitudinal studies of European or African descent. The BMES reported a hyperopic shift of 0.4 D for the 49–54 years old group, and a myopic shift of -0.02 D for the 65–74 years old group.[12] In the BDES, the 10 year refractive change for participants aged 43–84 years old showed hyperopic shifts for participants under the age of 70 and myopic shifts for participants over the age of 70.[11] The BES showed a median of 0.38 D hyperopic shift for individuals aged 40–49 years and a median of -0.75 D myopic shift for individuals aged ≥ 60 years.[13] The Reykjavik Eye study also reported a hyperopic shift for subjects younger than 70 years and a myopic shift thereafter.[14] However, the Shahroud Eye study reported a hyperopic shift in all age groups, and the absence of myopic shift might be due to the limited age range of participants.[15] The observed mean SE change in our analysis was comparable or slightly greater than the above studies and the absence of myopic shift among the older people in our previously 2-year analysis was mainly due to short follow-up period.[16]

The observed myopic shift in older subjects had been attributed to the development and progression of cataract.[21] Based on our analysis, the myopia shift in the 65+ age group turned into significant hyperopic shift if participants who had cataract at baseline or follow-ups were excluded (Tables 2 and 3). When analysis was performed separately for participants with baseline cataract and those without, the overall hyperopic shift in SE was greater in the latter group (S2 and S3 Tables). In addition, the percentage of people with a myopic change of more than -0.5 D during the follow-up was greater in participants with cataract at baseline than those without. Furthermore, more myopic SE at baseline was not related to the change in LP during the follow-up. All these results in our analysis strongly supports the myopia shift in older subjects was caused by cataract. Presence of baseline nuclear cataract was associated with myopic shift in some previous studies and a positive association between incident cataract and myopic refractive shift was also reported in the BMES.[5, 22] However, baseline cataract was associated with SE change only in the univariate regression in our study, the reason might be lack of cataract grading in our study and the effect of cataract on SE change was partially mediated by biometric factors.

It has been suggested that ocular refraction depends on the interaction of ocular biometric factors. We observed a small increase in corneal power, AL and LT during the follow-up as well as decrease in ACD and LP. The amount of decrease in ACD was roughly half of the increase in LT, which suggested a bi-directional thickening of the lens during cataract development and progression. In the multiple regression model, more myopic baseline SE, decrease in AL, corneal power, LT and LP were all significantly related to a hyperopic shift in SE and in total they could explain 97.2% of the variance in SE change. It could be inferred that, the balance between the myopic effect caused by increasing corneal power, AL and LT and the hyperopic effect caused by decreasing LP during the 6-year follow-up contributes to the potential mechanisms for the observed hyperopic and myopic shift. LT as well as anterior and posterior surface curvatures continue to increase with age. The observed overall hyperopic rather than myopic shift was known as lens paradox, and was suggested to result from the decrease in the gradient refractive index of the lens compensating for the increased surface curvatures.[23, 24]

Lens power was found to be the most important biometric index related to SE change. The greatest hyperopic shift was found in the 45 to 54 age group, which could be explained by the greatest decline of LP at these ages (Table 1). The only other study which assessed the association between longitudinal SE and biometry change was the Shahroud Eye Cohort Study, and they also found LP to be the most important factor. The 5-year changes in biometric factors in their study were smaller than ours, and even smaller than our previously reported 2-year changes, this may due to difference in population and measurement instruments. It should also be noted that both studies found myopes had larger increase in AL. The reason might be increased sclera fragility with more myopic SE, resulting in higher vulnerability to expand under the effect of intraocular pressure. And this also explains why myopes had less hyperopic shifts during the follow-up.

Education level was related to longitudinal SE change only in the univariate analysis, this suggested that the effect of education level on SE was mediated by biometric factors. Similar results were found in the BMES and BDES.[11, 12] Gender was found to be irrelevant with SE change in our study, which is consistent with the majority of previous studies except for the BES and Shahroud Eye Cohort Study which reported greater hyperopic shift in women. A higher prevalence of hyperopia in women has been observed in some studies, but not consistently.[25] Baseline refractive status was associated with SE change in our study as well as in the 10-year BDES and Shahroud Eye Cohort Study but not in the BMES, which may be explained by the difference in statistical models and variables included in the model.

We also observed a birth cohort effect on SE change when comparing people in different birth cohorts at the same age. This was also illustrated in other previous studies.[12, 16] Younger generations were found to be more myopic than older generations, possibly due to increased exposure to increased schooling intensity among the 35–40 years old people, secondary to the re-establishment of the education system in late 1970s in China.

Most longitudinal studies failed to report on the age-related changes in astigmatism. We observed minor decrease in mean cylinder power which is greatest in the oldest age group. This is consistent with the reported higher prevalence of astigmatism in older subjects.[26] With regards to the axis, there was a trend towards against-the-rule astigmatism with age. This is in agreement with cross-sectional findings and several other longitudinal studies.[27, 28] The Reykjavik Eye study found a mean change of 0.13 D in the against-the-rule astigmatism and the Blue Mountain Eye Study found a 10% increase in against-the-rule astigmatism over a 10-year period.[12, 14]

The results of our study offer practical information for planning eye care services for the aging society as well as predicting long-term outcomes for the ever-growing application of refractive surgery. As there is a relatively high prevalence of primary angle-closure glaucoma in the Chinese population, the longitudinal increase in LT and decrease in ACD, especially in younger and myopic subjects indicate that we should enhance glaucoma screening and pay more attention to this subset of population.[29] We could also speculate that, as subjects with more myopic SE have less hyperopic shifts, the prevalence of angle-closure glaucoma will decrease in China with the increasing prevalence of myopia. The limitations of our study include the absence of cataract classification and using different measurement tools for biometry measurement. Fortunately, all the tools were used at the 2010 follow-up with highly consistent values, which can be used to reliably convert the measurements from different tools for direct comparison.

In conclusion, we have documented the 6-year changes in SE and ocular biometry in an adult Chinese population. The change in SE showed a hyperopic shift for people aged 35–65 years old and a myopic shift thereafter. Longitudinal change in SE could be largely explained by baseline SE, changes in corneal power, AL, LT and especially LP. There was a small amount of change in astigmatism and an against-the-rule change of the axis. Further studies are needed to better understand this issue. Future spectacle correction, refractive surgery and glaucoma screening for adults should take these longitudinal changes in SE and ocular biometry into consideration.

## Supporting information

S1 Table Comparison of participants included and not-included in the analysis. (DOCX)

S2 Table Six-year changes of spherical equivalence and related biometric factors in the right eye of participants with cataract at baseline. (DOCX)

S3 Table Six-year changes of spherical equivalence and related biometric factors in the right eye of participants without cataract at baseline. (DOCX)

S1 Fig Spherical equivalent refraction at baseline and 6-year follow-up in different birth cohorts. (TIF)

S1 File Brief study questionnaire. (DOCX)
