# Supplementary material for: Six-year changes in refraction and related ocular biometric factors in an adult Chinese population
Source: PLoS One. 2017 Aug 30;12(8):e0183364. doi: 10.1371/journal.pone.0183364 (PMC5576680; doi:10.1371/journal.pone.0183364)
Supplement: S1 Table — (DOCX) [file pone.0183364.s001.docx]

Supplement Table1. Comparison of participants included and not-included in the analysis.

| **Baseline**  **Characteristic** | **Included** | | **Not-included** | | **P value** |
| --- | --- | --- | --- | --- | --- |
|  | **N** | **Mean ± SD** | **N** | **Mean ± SD** |  |
| Age, y | 1300 | 51.4 ± 10.6 | 517 | 58.3 ± 16.5 | <0.001 |
| Height, cm | 1280 | 160.3 ± 8.3 | 429 | 159.1 ± 8.6 | 0.01 |
| Weight, kg | 1244 | 61.7 ± 10.8 | 413 | 60.3± 11.0 | 0.03 |
| Baseline SE, D | 1300 | -0.4 ± 2.2 | 484 | -0.7 ± 2.8 | 0.08 |
| Corneal Power, D | 1295 | 43.8 ± 1.5 | 484 | 43.7 ± 1.6 | 0.30 |
| AL, mm | 676 | 23.6 ± 1.1 | 253 | 23.8 ± 1.3 | 0.03 |
| ACD, mm | 676 | 3.2 ± 0.4 | 256 | 3.2 ± 0.5 | 0.44 |
| LT, mm | 640 | 6.2± 0.5 | 210 | 6.3 ± 0.6 | 0.19 |
|  | **N** | **%** | **N** | **%** |  |
| Sex, female | 1300 | 54.5 | 517 | 52.6 | 0.46 |
| Baseline Cataract, yes | 1300 | 26.6 | 517 | 36.9 | <0.001 |
| Educational level, high school completed | 1300 | 68.2 | 517 | 58.8 | <0.001 |

SE: spherical equivalence; AL: axial length; ACD: anterior chamber depth; LT: lens thickness; D: diopter; SD: standard deviation.
