# Supplementary material for: Six-year changes in refraction and related ocular biometric factors in an adult Chinese population
Source: PLoS One. 2017 Aug 30;12(8):e0183364. doi: 10.1371/journal.pone.0183364 (PMC5576680; doi:10.1371/journal.pone.0183364)
Supplement: S2 Table — (DOCX) [file pone.0183364.s002.docx]

Supplement table2. Six-year changes of spherical equivalence and related biometric factors in the right eye of participants with cataract at baseline.

| **Characteristic** | **SE**  **(mean, 95% CI),D** | **Corneal power**  **(mean, 95% CI),D** | **ACD**  **(mean, 95% CI),mm** | **LT**  **(mean, 95% CI),mm** | **LP**  **(mean, 95% CI),D** |
| --- | --- | --- | --- | --- | --- |
| Total No. | 346 | 334 | 161 | 152 | 148 |
| Difference | 0.14(0.04 to 0.25) | 0.25(0.22 to 0.29) | -0.05(-0.07 to -0.02) | 0.09(0.06 to 0.13) | -1.24(-1.52 to -0.96) |
| **Age group** |  |  |  |  |  |
| 35-44 | 0.13(-1.46 to 1.71) | 0.13(0.13 to 0.13) | 0.18 | 0.02 | -0.74 |
| 45-54 | 0.41(0.21 to 0.60) | 0.31(0.25 to 0.37) | -0.08(-0.13 to -0.03) | 0.09(-0.04 to 0.22) | -2.02(-2.39 to -1.65) |
| 55-64 | 0.21(0.03 to 0.38) | 0.23(0.17 to 0.29) | -0.08(-0.10 to -0.05) | 0.11(0.08 to 0.14) | -1.17(-1.55 to -0.78) |
| ≥65 | -0.04(-0.22 to 0.13)^b^ | 0.25(0.19 to 0.32) | 0.01(-0.02 to 0.05)^b^ | 0.07(-0.01 to 0.16)^b^ | -0.93(-1.51 to -0.36)^b^ |
| **Sex** |  |  |  |  |  |
| Male | 0.24(0.07 to 0.40) | 0.24(0.18 to 0.30) | -0.05(-0.08 to -0.01) | 0.12(0.08 to 0.17) | -1.50(-1.94 to -1.07) |
| Female | 0.06(-0.07 to 0.20) | 0.26(0.22 to 0.31) | -0.04(-0.07 to -0.02) | 0.07(0.00 to 0.13) | -0.97(-1.30 to -0.63) |
| **Education** |  |  |  |  |  |
| Less than high school | 0.08(-0.06 to 0.21) | 0.28(0.23 to 0.33) | -0.03(-0.06 to -0.00) | 0.05(-0.02 to 0.11) | -1.14(-1.56 to -0.72) |
| High school or above | 0.22(0.05 to 0.38) | 0.23(0.17 to 0.28) | -0.06(-0.09 to -0.03) | 0.14(0.10 to 0.19)^a^ | -1.34(-1.71 to -0.98) |
| **Baseline refractive state** |  |  |  |  |  |
| Moderate to high myopia | 0.05(-0.33 to 0.42) | 0.30(0.21 to 0.40) | -0.01(-0.10 to 0.09) | 0.03(-0.20 to 0.27) | -1.95(-3.48 to -0.41) |
| Mild myopia | -0.21(-0.70 to 0.27) | 0.28(0.18 to 0.39) | -0.06(-0.11 to -0.01) | 0.07(0.00 to 0.14) | -0.18(-1.32 to 0.95) |
| Emmetropia | 0.22(0.03 to 0.41) | 0.25(0.16 to 0.33) | -0.06(-0.10 to -0.02) | 0.10(0.03 to 0.17) | -1.49(-1.80 to -1.17) |
| Hyperopia | 0.22(0.10 to 0.33) | 0.24(0.19 to 0.29) | -0.04(-0.07 to -0.01) | 0.11(0.06 to 0.16) | -1.26(-1.57 to -0.96) |

SE: spherical equivalence; ACD: anterior chamber depth; LT: lens thickness; LP: lens power; D: diopter; CI: confidence interval.

^a^ P ≤ 0.05; ^b^ P ≤ 0.001
