# Supplementary material for: Six-year changes in refraction and related ocular biometric factors in an adult Chinese population
Source: PLoS One. 2017 Aug 30;12(8):e0183364. doi: 10.1371/journal.pone.0183364 (PMC5576680; doi:10.1371/journal.pone.0183364)
Supplement: S3 Table — (DOCX) [file pone.0183364.s003.docx]

Supplement table3. Six-year changes of spherical equivalence and related biometric factors in the right eye of participants without cataract at baseline.

| **Characteristic** | **SE**  **(mean, 95% CI),D** | **Corneal power**  **(mean, 95% CI),D** | **ACD**  **(mean, 95% CI),mm** | **LT**  **(mean, 95% CI),mm** | **LP**  **(mean, 95% CI),D** |
| --- | --- | --- | --- | --- | --- |
| Total No. | 954 | 939 | 436 | 414 | 406 |
| Difference | 0.38(0.34 to 0.42) | 0.29(0.27 to 0.31) | -0.07(-0.08 to -0.06) | 0.17(0.15 to 0.18) | -1.76(-1.86 to -1.66) |
| **Age group** |  |  |  |  |  |
| 35-44 | 0.24(0.19 to 0.30) | 0.30(0.27 to 0.34) | -0.09(-0.11 to -0.08) | 0.22(0.19 to 0.24) | -1.79(-1.95 to -1.62) |
| 45-54 | 0.53(0.48 to 0.58) | 0.28(0.25 to 0.31) | -0.05(-0.07 to -0.03) | 0.15(0.13 to 0.17) | -1.92(-2.01 to -1.83) |
| 55-64 | 0.32(0.18 to 0.47) | 0.24(0.20 to 0.28) | -0.07(-0.10 to -0.04) | 0.11(0.09 to 0.13) | -1.33(-1.69 to -0.97) |
| ≥65 | -0.11(-0.52 to 0.30)^b^ | 0.31(0.22 to 0.40) | -0.08(-0.16 to -0.01)^a^ | 0.13(-0.04 to 0.30)^b^ | -0.64(-2.22 to 0.94) |
| **Sex** |  |  |  |  |  |
| Male | 0.39(0.33 to 0.45) | 0.29(0.26 to 0.32) | -0.07(-0.09 to -0.05) | 0.17(0.15 to 0.19) | -1.76(-1.89 to -1.63) |
| Female | 0.37(0.31 to 0.42) | 0.28(0.25 to 0.31) | -0.07(-0.09 to -0.05) | 0.17(0.15 to 0.19) | -1.76(-1.90 to -1.62) |
| **Education** |  |  |  |  |  |
| Less than high school | 0.38(0.30 to 0.45) | 0.28(0.25 to 0.31) | -0.06(-0.08 to -0.04) | 0.15(0.13 to 0.17) | -1.61(-1.83 to -1.39) |
| High school or above | 0.38 (0.33 to 0.42) | 0.29(0.26 to 0.31) | -0.07(-0.09 to -0.06) | 0.18(0.16 to 0.19) | -1.81(-1.92 to -1.71) |
| **Baseline refractive state** |  |  |  |  |  |
| Moderate to high myopia | 0.003(-0.24 to 0.25) | 0.22(0.08 to 0.36) | -0.11(-0.15 to -0.07) | 0.22(0.19 to 0.25) | -1.70(-2.34 to -1.06) |
| Mild myopia | 0.27(0.18 to 0.35) | 0.30(0.27 to 0.34) | -0.07(-0.11 to -0.03) | 0.19(0.16 to 0.22) | -1.64(-1.86 to -1.43) |
| Emmetropia | 0.45(0.41 to 0.48) | 0.29(0.27 to 0.31) | -0.07(-0.08 to -0.06) | 0.17(0.15 to 0.19) | -1.82(-1.91 to -1.73) |
| Hyperopia | 0.53(0.45 to 0.61)^b^ | 0.29(0.25 to 0.33) | -0.04(-0.06 to -0.02)^b^ | 0.12(0.10 to 0.15)^b^ | -1.76(-1.99 to -1.53)^a^ |

SE: spherical equivalence; ACD: anterior chamber depth; LT: lens thickness; LP: lens power; D: diopter; CI: confidence interval.

^a^ P ≤ 0.05; ^b^ P ≤ 0.001
