# Supplementary figures and images for: Six-year changes in refraction and related ocular biometric factors in an adult Chinese population
Source: PLoS One. 2017 Aug 30;12(8):e0183364. doi: 10.1371/journal.pone.0183364 (PMC5576680; doi:10.1371/journal.pone.0183364)

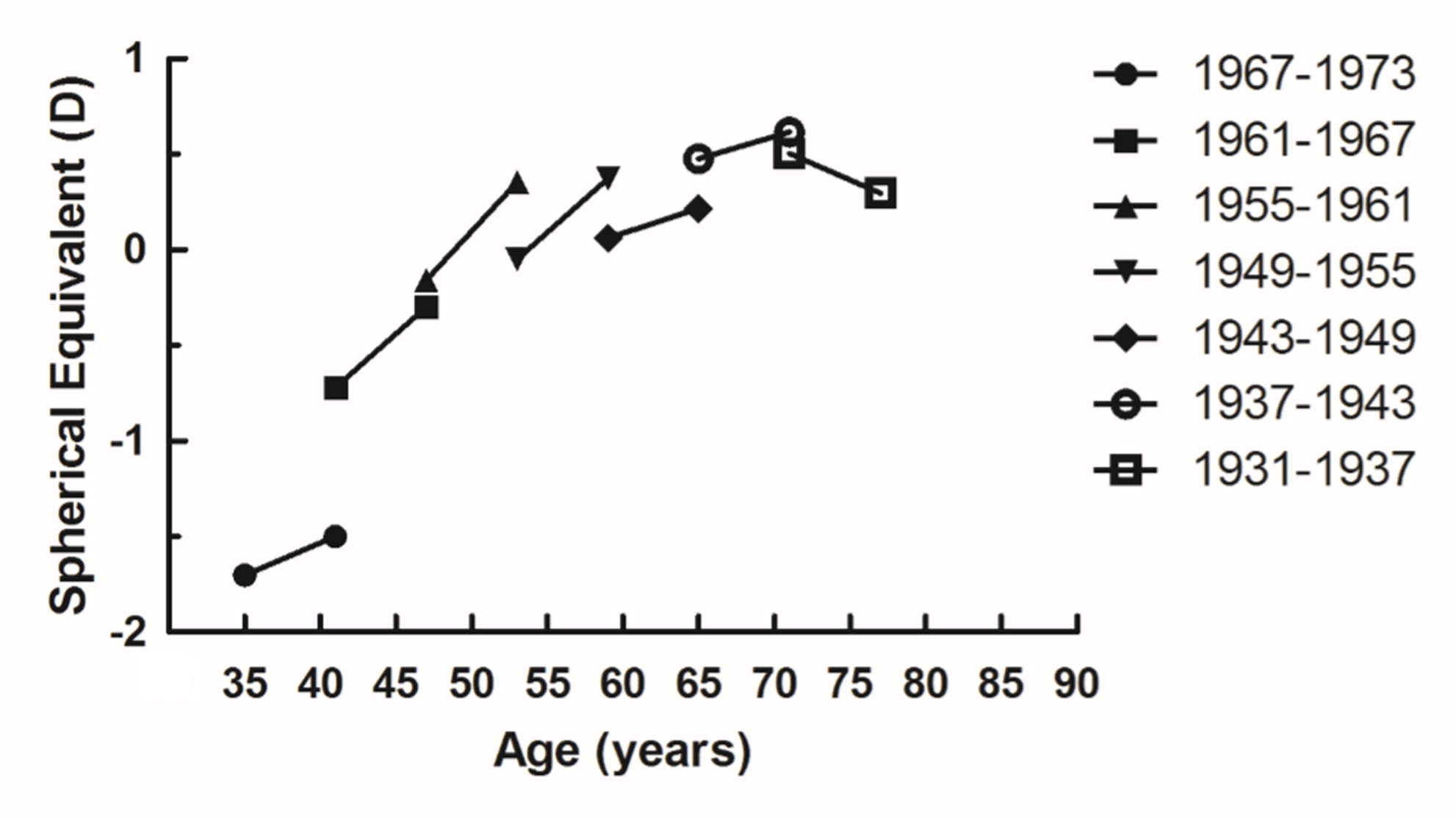

Supplement: S1 Fig — (TIF) [file pone.0183364.s004.tif]
