# Supplementary material for: Six-year changes in refraction and related ocular biometric factors in an adult Chinese population
Source: PLoS One. 2017 Aug 30;12(8):e0183364. doi: 10.1371/journal.pone.0183364 (PMC5576680; doi:10.1371/journal.pone.0183364)
Supplement: S1 File — (DOCX) [file pone.0183364.s005.docx]

基础数据表

**第一步 登记**

**A1.**

受检者ID: ________________ 姓名：__________________

电话：__________________ 住址：_________________

籍贯：________________ 检查时间：（日-月-年）____-____-___

生日: ___/___/___(mm/dd/yy) 性别(1.男； 2.女):

检查地点：

1. 家里；2. 社区；3. 医院

**A2.** 以前是否做过眼部手术?

0. 否； 1. 准分子近视手术；

2. 白内障手术； 3. 青光眼手术；

4. 内眼手术； 5. 其他,请说明________________

**A3.**以前是否患过重病？(1:是； 2:否):

BASIC DATA FORM

**Section1**

**A1.**

ID: ________________ Name：__________________

Tele：__________________ Address：_________________

Birthplace：_____________ Date: (dd-mm-yy) ____-____-___

Birthday: ___/___/___ (mm/dd/yy) Sex (1.Male; 2. Female):

Measurement Site：

1. Home; 2. Community Site; 3. Clinic/hospital

**A2.** Have you ever undergone any kind of eye surgeiry?

0. None; 1. Refractive Laser Surgery;

2. Cataract Surgery; 3. Glaucoma surgery;

4. Inner eye surgery; 5. Others, please specify________________

**A3.** Have your ever had severe systemic disease？(1: Yes; 2: No):
